# Yogaman: An Inexpensive, Anatomically-detailed Chest Tube Placement Trainer

**DOI:** 10.5811/westjem.2018.9.39456

**Published:** 2018-11-20

**Authors:** Timothy P. Young, Mark D. Schaefer, Heather M. Kuntz, Molly K. Estes, Michael Kiemeney, Brian J. Wolk, Mindi Guptill

**Affiliations:** Loma Linda University Medical Simulation Center, Loma Linda, California, Loma Linda University Medical Center, Department of Emergency Medicine, Loma Linda, California

## Abstract

**Introduction:**

Opportunities for chest tube placement in emergency medicine training programs have decreased, making competence development and maintenance with live patients problematic. Available trainers are expensive and may require costly maintenance.

**Methods:**

We constructed an anatomically-detailed model using a Halloween skeleton thorax, dress form torso, and yoga mat. Participants in a trial session completed a survey regarding either their comfort with chest tube placement before and after the session or the realism of Yogaman vs. cadaver lab, depending on whether they had placed <10 or 10 or more chest tubes in live patients.

**Results:**

Inexperienced providers reported an improvement in comfort after working with Yogaman, (comfort before 47 millimeters [mm] [interquartile ratio {IQR}, 20–53 mm]; comfort after 75 mm [IQR, 39–80 mm], p=0.01). Experienced providers rated realism of Yogaman and cadaver lab similarly (Yogaman 79 mm [IQR, 74–83 mm]; cadaver lab 78 mm [IQR, 76–89 mm], p=0.67). All evaluators either agreed or strongly agreed that Yogaman was useful for teaching chest tube placement in a residency program.

**Conclusion:**

Our chest tube trainer allowed for landmark identification, tissue dissection, pleura puncture, lung palpation, and tube securing. It improved comfort of inexperienced providers and was rated similarly to cadaver lab in realism by experienced providers. It is easily reusable and, at $198, costs a fraction of the price of available commercial trainers.

## BACKGROUND

As the number of learners in teaching institutions increases, live procedure opportunities are on the decline.^1^ This trend is especially concerning when uncommon procedures are considered. Chest tubes in emergency departments are becoming less common, and patterns of chest tube placement are evolving. Clinical practice has transitioned toward smaller-diameter Seldinger chest tube placement and observational management of small pneumothoraces.^2^ Chest tube placement is associated with serious complications, including placement into the liver and chest wall, disruption of the diaphragm, injury to the lung, and damage to the intercostal neurovascular bundle.

Faculty at teaching institutions may be hesitant to allow learners with no practical experience to place a chest tube in a critical situation, when time is short and the risk of complications is high. Opportunities to learn and practice the procedure outside of live patient care settings are therefore important. Simulation models, when part of a structured approach to procedural learning, are one potential solution.^3^ In our training program, we did not previously have a good option for simulating chest tube placement outside of our yearly cadaver lab. Current simulation models that allow chest tube placement are some of the most expensive on the market, ranging from thousands to tens of thousands of United States dollars.^4^–^6^ Animal-based models have been proposed as a less expensive option, but are messy and not reusable.^7^ These models simulate only the chest wall and do not provide human anatomic landmarks or simulate the lungs.

## OBJECTIVES

We constructed a do-it-yourself chest tube model built around a Halloween skeleton, plastic dress-form torso, and yoga mat. We named the trainer Yogaman. We evaluated the model’s realism and effect on provider comfort with chest tube placement in a trial session. We were considering ending our yearly cadaver lab, and were interested to see how perceived realism compared with chest tube placement in cadaver lab.

## CURRICULAR DESIGN

### Model Construction

**Figure 1:**
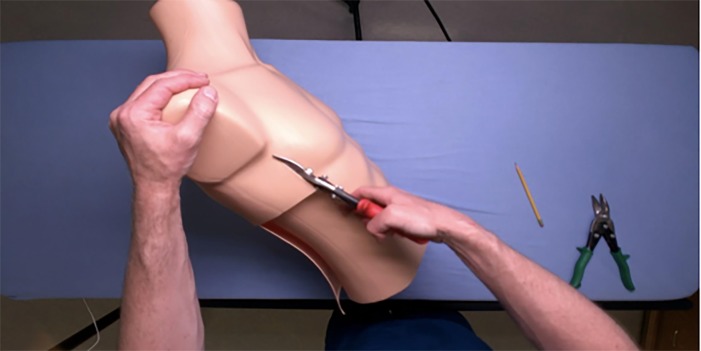
Cutting the dress form torso with tin snips to create the Yogaman chest tube trainer.

Construction of the model is straightforward. The only tool needed is a heavy-duty cutting device. We used tin snips, but a sturdy pair of trauma shears will also work. All of the materials were purchased on amazon.com, and the total cost was $198 (Table). The plastic dress-form must be cut to allow for the insertion of the skeleton thorax (Figure 1). We marked the cuts in pencil prior to cutting. We made cuts to expose the “triangle of safety” on either side of the thorax. The yoga mat must be folded in half and cut, and then cut again lengthwise into six-inch strips to simulate the chest wall. One yoga mat supplies eight chest-wall strips. The memory foam mattress pad is cut in a 10-inch strip and rolled to the center from both ends to simulate lungs.

**Figure 2:**
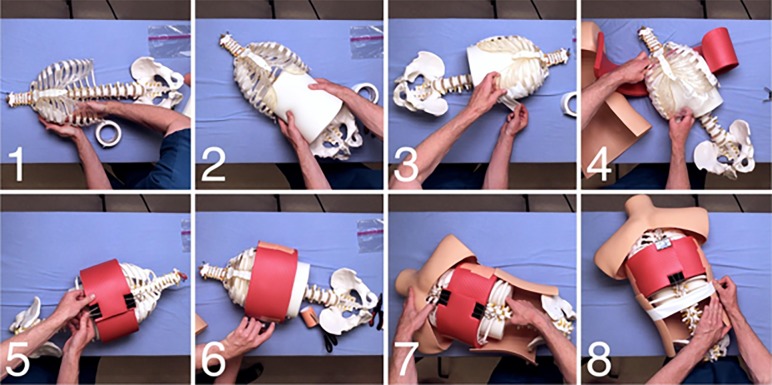
Yogaman assembly. The eight-step Yogaman assembly process. 1) Line the inside of the upper thorax with gaffer tape to allow the tube to slide along the ribs once in the pleural space. 2) Insert the memory foam lungs. 3) Place gaffer tape on the area to be punctured. 4) Insert the resealable sandwich bag to act as a pneumothorax (optional). 5) Wrap the chest wall and clamp with binder clips. 6) Place pink surgical tape to act as skin on the lateral chest wall. 7) Place the thorax into cut plastic, dress-form torso. 8) Cinch the dress form to the thorax with gaffer tape.

Assembly of Yogaman is an eight-step process (Figure 2). In use, the model allows learners to make the initial skin incision, bluntly dissect the chest wall, puncture the pleura, and advance the chest tube to the desired location. The yoga mat chest wall can be sutured. Gaffer tape can be used as a less-expensive substitute for chest tube tape so that learners can practice securing the tube, and has the additional benefit that it does not leave behind residue. Once tube placement is complete, the model can be taken apart to determine exact tube placement. The chest wall can be rotated until there are too many cuts to reuse it, at which time a new chest wall is placed. Yogaman takes approximately two minutes to assemble. We created a video that demonstrates the construction and assembly of Yogaman in greater detail and posted it on YouTube (https://youtu.be/TtNIqda4x_8). Links to materials appear on the page.

### Assessment

We trialed Yogaman with a group of practitioners of varying experience after an educational meeting. The group consisted of 18 emergency medicine (EM) residents and faculty. Aside from open thoracostomy tube placement, the model also allowed for Seldinger chest tube placement and needle thoracostomy practice. The yoga mat allowed for the application of negative pressure during needle insertion and location of the pleural space. We discovered that a resealable sandwich bag can be placed between the lung (rolled memory foam mattress topper) and chest wall to make needle decompression of a tension pneumothorax audible. The mattress topper places the sandwich bag under pressure, which results in a pop and rush of air when the “pleura” is punctured. Yogaman can be placed on a gurney or table; we prefer an adjustable-height bedside table. Because the model is light, we find it helpful for observers to hold the model down. Alternatively, gaffer tape run from one side of the gurney or table across Yogaman’s waist to the other side can also act as an anchor. We created a second YouTube video to demonstrate Yogaman in use (https://youtu.be/oLp74QCKBgw).

After the session, we asked the attendees to complete an evaluation. Separate evaluations were created for practitioners who had performed less than 10 (“inexperienced”) or 10 or more (“experienced”) chest tubes in live patients. We chose 10 because that is the number the Accreditation Council for Graduate Medical Education Emergency Medicine Residency Review Committee requires for graduates of EM training programs. 8 The experienced evaluation asked the evaluator to rate the realism of Yogaman on a 100-millimeter (mm) visual analog scale (VAS), anchored with “not realistic at all” on the left and “as realistic as possible” on the right. It then asked if the participant had placed a chest tube in cadaver lab. If so, the participant was asked to rate the realism of cadaver lab for chest tube placement on a VAS. For participants who had placed fewer than 10 chest tubes in live patients, the survey asked for a rating of comfort in placing a chest tube before working with Yogaman on a 100-mm VAS, and comfort after the session on a 100-mm VAS. This scale was anchored with “not comfortable at all” on the left and “as comfortable as possible” on the right. All evaluators were asked to rate agreement with the statement “Yogaman is useful for teaching chest tube placement in a residency program” on a five-point Likert scale (“Strongly Disagree” to “Strongly Agree”). We gave participants a gift certificate for a beverage from a campus café as appreciation for completing the survey.

## IMPACT/EFFECTIVENESS

Of 18 meeting attendees, 16 completed an evaluation (89%). We compared comfort before and after using a Wilcoxon matched-pairs test, and compared realism with a Mann-Whitney test. Inexperienced providers (N=8) reported an improvement in comfort after working with Yogaman (median comfort before 47 mm [IQR, 20–53 mm]; median comfort after 75 mm [IQR, 39–80 mm], p=0.01). All participants who had placed 10 or more chest tubes in live patients (N=8) had also placed a chest tube in cadaver lab. These providers rated the realism of Yogaman and cadaver lab similarly (median realism of Yogaman 79 mm [IQR, 74–83 mm]; median realism of cadaver lab 78 mm [IQR, 76–89 mm], p=0.67). All evaluators either agreed or strongly agreed that Yogaman was useful for teaching chest tube placement in a residency program.

Prior to creating this model, we were challenged to allow our residents to practice chest tube placement in a simulation environment. Our program consists of over 40 residents, and we hold bimonthly skills labs with up to half of our residency complement working on one procedure simultaneously. Multiple models are a necessity to minimize downtime, which makes the use of commercial models cost prohibitive. To date, we have created and used three models with our EM trainees.

Previously, cadaver lab was our primary modality for chest tube teaching. We experienced several limitations with this strategy. Each cadaver is limited in the number of times a chest tube can be placed. Also, the lab is time intensive to arrange and costly; hence, we were not able to offer the teaching activity more than once per year. This made repeat practice difficult. Yogaman solved these problems. Due in part to the success of Yogaman, we plan to end our yearly cadaver lab.

We find that live chest tube experience varies from trainee to trainee. Occasionally, those who feel they have not had enough opportunities to perform live chest tube placement will ask for addition simulation time, and until now we did not have a good option for practice. Now, if a trainee has difficulty or would simply like more practice, we are able to offer additional simulation opportunities with Yogaman. In this way, his lower cost and portability allows us to deliver teaching and practice when and where it is needed.

Our evaluation of Yogaman was intended to be a pilot demonstration of the feasibility of our do-it-yourself model. Accordingly, there were some limitations. While validity evidence exists for the VAS in the settings of self-reported pain,^9^ anxiety,^10^ and quality of life,^11^ it does not exist for confidence with a technical skill. Similarly, our choice of wording for the assessment of learner achievement was not ideal. The term “confidence” is preferable to “comfort” when measuring the learner’s self-assessment of achievement in situations that involve a complicated, high-risk procedure. We would hope that no practitioner becomes comfortable with inserting a chest tube for fear that they will become complacent or non-vigilant.

## CONCLUSION

Our chest tube model was much less expensive than commercial trainers and allowed complete performance of a critical emergency procedure. It was well-received by a group of faculty and residents. Other training programs may wish to use Yogaman to increase flexibility in teaching, learning and practicing chest tube placement.

## Figures and Tables

**Figure 3:**
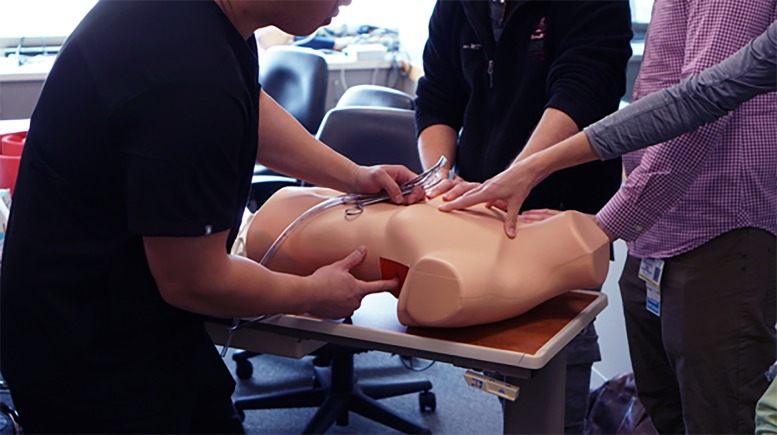
The Yogaman chest tube model in use.

**Table:** List of materials for the chest tube placement model.

Material	Anatomy/function	Price paid
5/8″ red yoga mat	Chest wall	$18
3″ pink surgical tape	Skin	$14
Plastic skeleton torso	Ribs	$83
White gaffer tape	Pleura	$15
Torso dress form	External landmarks	$25
1″ memory foam mattress topper	Lungs	$12
2″ binder clips	Fastener	$9
Resealable sandwich bags	Pneumothorax	$4
	Total	$198

## References

[1] Gisondi MA, Regan L, Branzetti J (2018). More learners, finite resources, and the changing landscape of procedural training at the bedside. Acad Med.

[2] Martin K, Emil S, Zavalkoff S (2013). Transitioning from stiff chest tubes to soft pleural catheters: prospective assessment of a practice change. Eur J Pediatr Surg.

[3] Davis AJ, Fierro L, Guptill M (2017). Practical application of educational theory for learning technical skills in emergency medicine. Ann Emerg Med.

[4] Nasco, Life/form® Chest Tube Manikin.

[5] TruCorp TruMan Trauma X: Surgical Airway Management & Resuscitation Skills.

[6] Simulab Corporation, Simulab Corporation Life or Death Decision: TraumaMan Offers Humane, Effective Way to Learn Trauma Care.

[7] Van Doormaal CJ, Howes DW, Salazar CL (2011). 112 An Innovative and inexpensive pork ribs model for teaching tube thoracostomy. Ann Emerg Med.

[8] Accreditation Council for Graduate Medical Education Review Committee for Emergency Medicine Emergency Medicine Defined Key Index Procedure Minimums.

[9] Scott J, Huskisson EC (1976). Graphic representation of pain. Pain.

[10] Williams VS, Morlock RJ, Feltner D (2010). Psychometric evaluation of a visual analog scale for the assessment of anxiety. Health Qual Life Outcomes.

[11] de Boer AGEM, van Lanschot JJ, Stalmeier PF (2004). Is a single-item visual analogue scale as valid, reliable and responsive as multi-item scales in measuring quality of life?. Qual Life Res.

